# Collagen fibrils and proteoglycans of peripheral and central stroma of the keratoconus cornea - Ultrastructure and 3D transmission electron tomography

**DOI:** 10.1038/s41598-019-56529-1

**Published:** 2019-12-27

**Authors:** Aljoharah Alkanaan, Robert Barsotti, Omar Kirat, Adnan Khan, Turki Almubrad, Saeed Akhtar

**Affiliations:** 1 Cornea Research Chair, Department of Optometry, College of Applied Medical Sciences, King Saud University, Riyadh, Saudi Arabia; 2 Department of Biomedical Sciences, Philadelphia college of Osteopathic Medicine, Philadelphia, PA USA; 3 Department of Ophthalmology, King Khalid Eye Specialist Hospital, Riyadh, Saudi Arabia

**Keywords:** Cells, Translational research

## Abstract

Keratoconus (KC) is a progressive corneal disorder in which vision gradually deteriorates as a result of continuous conical protrusion and the consequent altered corneal curvature. While the majority of the literature focus on assessing the center of this diseased cornea, there is growing evidence of peripheral involvement in the disease process. Thus, we investigated the organization of collagen fibrils (CFs) and proteoglycans (PGs) in the periphery and center of KC corneal stroma. Three-dimensional transmission electron tomography on four KC corneas showed the degeneration of microfibrils within the CFs and disturbance in the attachment of the PGs. Within the KC corneas, the mean CF diameter of the central-anterior stroma was significantly (p ˂ 0.001) larger than the peripheral-anterior stroma. The interfibrillar distance of CF was significantly (p ˂ 0.001) smaller in the central stroma than in the peripheral stroma. PGs area and the density in the central KC stroma were larger than those in the peripheral stroma. Results of the current study revealed that in the pre- Descemet’s membrane stroma of the periphery, the degenerated CFs and PGs constitute biomechanically weak lamellae which are prone to disorganization and this suggests that the peripheral stroma plays an important role in the pathogenicity of the KC cornea.

## Introduction

The high transparency and regular curvature of the corneal tissue enables it to serve its refractive function efficiently. These essential corneal properties: transparency and curvature, are dependent upon the organization of stromal lamellae and the arrangement of Collagen fibrils (CFs) within those lamellae^1–3^. The formation and organization of CFs are regulated by the proteoglycans (PGs) of the extracellular matrix^4^. It is suggested that in humans, the defective synthesis of stromal PGs is associated with alterations in fibrillar size, spacing and lamellar organization^5,6^. This results in either reduced corneal transparency or altered corneal curvature, and in any case a consequent impairment of vision^5,6^.

Keratoconus (KC) is a progressive corneal disease in which the vision gradually deteriorates as a result of continuous conical protrusion and the consequence altered corneal curvature^7^. Alteration in the corneal curvature observed in KC could be due to the changes in stromal biomechanics, which is believed to be stabilized by lamellar interconnectivity and the arrangement of collagen fibrils (CFs) within the lamellae^1,2^. Ultrastructural studies on the KC cornea have revealed disruption in the organization of CFs and alterations in PGs expression^8–14^.

Alterations in fibril arrangement and PGs expression were mainly assessed in the center of KC cornea however clinical studies provided evidence of peripheral thinning in the KC cornea which suggest alterations in the peripheral lamellar organization^15,16^. An assessment by Alkannan *et al*.^17^ of the stromal lamellae in the periphery of KC cornea revealed the presence of multiple undulating lamellae with disorganized CFs in the deep stromal layers. These observations lead us to consider the possibility of involvement of the peripheral stromal CFs and PGs in the process of the disease. We hypothesize that the involvement of the CFs and PGs in the peripheral stroma in the pathogenicity of the KC cornea which results slippage and degeneration of the lamellar arrangement.

## Material and Methods

**Figure 1:**
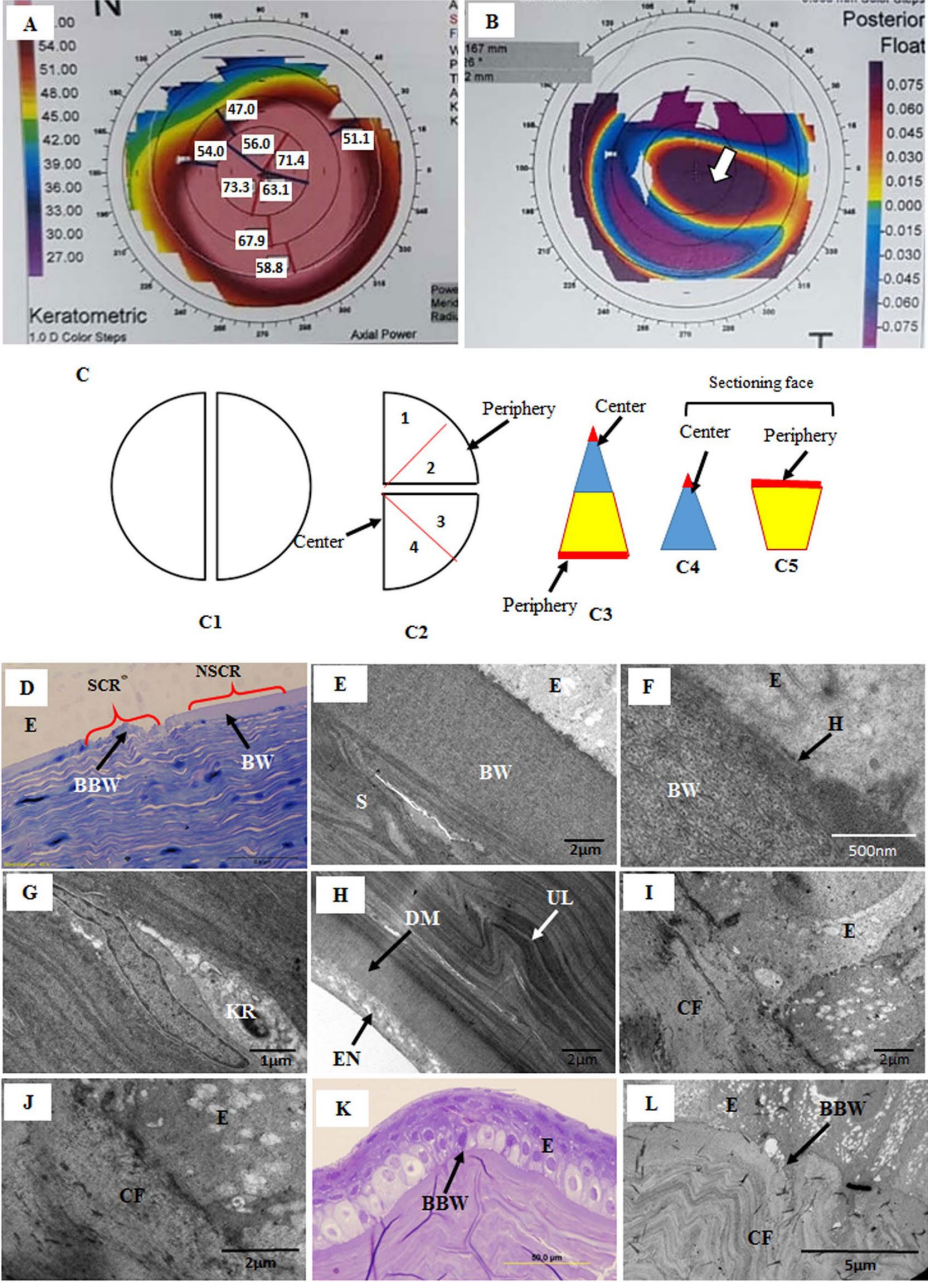
(**A**) Corneal topography axial map showing the extent of the cone (red area); (**B**) Corneal topography posterior elevation map showing the location of the cone (arrow head); (**C**) A schematic drawing of the cutting corneal central and peripheral pieces; (**1C1**) The cornea was divided into two halves; (**1C2**) Before embedding, the halve of the cornea was divided into two quarters and each quarter was further divided into equal triangle; (**1C3**) Each triangular piece was cut from the middle into two pieces; (**1C4**) the conical central part; and (**1C5**) wider peripheral part. Approximately less than 2 mm distance (red colour) from center point and approximately less than 1 mm distance (red colour) from the margin was used to cut semithin (0.5 µm) and ultrathin (70 nm) sections. (**D**) Light micrograph of keratoconus cornea showing non-scarred region (NSCR) with a normal Bowman’s layer (BW) and scarred region (SCR) with a break in BW; (**E,F**) Electron micrograph of non-scarred region (NSCR) shown in Figure A, a normal healthy BW, hemi-desmosome and stroma; (**G**) Electron micrograph of non-scarred region showing a healthy keratocyte; (**H**) At the posterior part of the non-scarred region, undulation of the lamellae was present just above the Descemet’s membrane; (**I,J**) Electron micrograph of scar region (SCR) shown in Figure A, showing break in BW and presence of collagen fibrils below epithelium; (**K**) Light micrograph of a very large scar region with a BW break; **L**) Electron micrograph of a large scar region (shown in Figure J), showing breaks in the BW and the presence of CF in the sub-epithelial region. E = Epithelium, BW = Bowman’s layer, BBW = Break in BW, H = Hemi-desmosomes, KR = Keratocyte, S = Stroma, SCR = Scar region, NSCR = Non-scar region.

Four human keratoconus cornea buttons of 8 mm diameter from subjects (aged from 24 years to 26 years; KC grade 3 and 4) were obtained from the surgeon of King Khalid Specialist Hospital. The surgeon marked the 12′oclock position on the cornea using a blue marker prior to removing the cornea. On the basis of topographic images of the cornea (Fig. 1A,B), the location and extension of the cone was identified on the cornea, and the conical area was marked by drawing a circle around it. The scarred and non-scarred region was further confirmed by light and electron microscopy (Fig. 1D,K).

The cornea was divided into two halves (Fig. 1C1). The first half of the cornea was fixed in 2.5% glutaraldehyde and osmium tetroxide to analyze the collagen fibril diameter and interfibrillar spacing^19^. The second half was fixed in 2.5% glutaraldehyde containing 0.05% cuprolinic blue (BDH Ltd, Poole, UK) using a critical electrolyte concentration mode^18^ to analyze the proteoglycans. The tissue were dehydrated into a graded series of ethanol (50% to 100%) and acetone^19^.

Each half of the cornea was divided into two quarters (Fig. 1C2) and each quarter was further divided into an equal size triangle (Fig. 1C2) and each triangular piece was cut from the middle to make two pieces (Fig. 1C3); the conical central part (Fig. 1C4) and the wider peripheral part (Fig. 1C5). The peripheral and central part were processed separately in individual small glass vials. The tissue was infiltrated into spurr resin for 8hrsX3.

These pieces were embedded into the resin facing conical and peripheral part upwards in separate block. The tissue were polymerized in resin for 8 hours at 70 °C to make blocks for semi-thin and ultra-thin sections. Four corneal normal buttons of the same size (subjects age 25 to 65 years) were processed with a similar procedure.

Eight blocks (4 blocks of ‘glutaraldehyde + osmium’ and 4 blocks of cuprolinic fixation) from each cornea were prepared. Six blocks of each cornea (3 blocks of ‘glutaraldehyde + osmium’ and 3 blocks of cuprolinic fixation) were cut to obtain semi-thin and ultra-thin sections. Approximately less than 2 mm distance (red colour) from the center point and approximately less than 1 mm distance (red colour) from the margin of the cornea was cut to get semi-thin (0.5 µm) and ultra-thin (70 nm) sections by using an RMC microtome.

From each keratoconus and normal cornea block, ten very good ultra-thin sections were cut and collected on 200 mesh copper grids. In total 24 KC blocks were cut and 240 sections were collected on grids. The semi-thin sections were stained with toluidine blue and observed with an Olympus BX54 light microscope.’ The JEOL 1400 (Jeol Ltd, Akishima, Japan) transmission electron microscope (TEM) is equipped with an 11 mega pixel Quemesa camera which was used to observe the ultra-thin sections which had been stained with 2% uranyl acetate (10 min) and lead citrate (10 min). From each section, 12 images were taken for CF measurements (total images 120) and 12 good images for PGs analysis (total images 120). All the images were taken from the same location with reference to the square of the 200 mesh grids. Approximately 720 images per cornea (2160 images per 3 corneas) were captured for analysis of CFs and PGs. In addition, a further 100 very good images were collected to observe the ultrastructure and to construct 3D images.

An 11 megapixel bottom-mounted Quemesa camera was used to capture digital images, using iTEM software^19^. In total, 120 digital images were taken from angles of −60° to +60° to construct individual 3D images using the software program “Composer-x64, version 3.4.2.0”. The color coding of 3D images were carried out by two methods described by Akhtar *et al*.^18^. To show the degradation of CF microfibrils, the color coding was done automatically by the program based on the electron density of the particles in the electron micrographs. To describe the PGs, the layers of images were color coded manually in a “Region of Interest” (ROI). The CFs were coded with blue in ten layers of the 3D image and these layers were merged into one image as “Image 1”^19^. The PGs of the same 3D image were not colored. The PGs were demonstrated as white color dots in the 3D image. ‘Image 1′ and the ‘3D image’ with white color areas indicated as PGs, were merged together. This method demonstrated the presence of PGs both in the surrounding matrix and on the CFs^20^.

The CFs diameter, CFs interfibrillar spacing, PGs area, PGs density and PGs coefficient variance (CV) were measured using the iTEM program. Data were analysed using the SPSS statistical software Version 18 after exporting them on to Excel spreadsheets from the imaging software. The mean CFs diameter and spacing, and PGs density and area of the anterior, middle and posterior stroma at the centre and periphery of the normal and KC cornea were calculated and then compared using the Mann-Whitney and Wilcoxon tests. Variation in the PGs area distribution between normal and KC corneas was assessed using Chi-square test.

### Ethical statement

Tissue procurement and use was ethically approved by the Local Ethical Committee; King Saud University, Saudi Arabia. All experiments were done in accordance with the guidelines of ‘Standing Committee for Research Ethics on Living Creatures (SCRELC)’ Saudi Arabia. Informed consent was obtained from all patients and tissue procurement and use was ethically approved by the Local Ethical Committee; King Saud University, Saudi Arabia.

Policy available at:

https://www.uod.edu.sa/sites/default/files/resources/implementing_regulations_0.pdf

## Results

### Light and electron microscopy features

Light and electron microscopy showed that the non-scared region has intact Bowman’s layer, basement membrane, hemi-desmosomes and keratocytes (Fig. 1D–G). At the posterior part of the non-scared region, undulation of the lamellae was present just above the Descemet’s membrane (Fig. 1H). In the scared region, Bowman’s break were observed and it was replaced by collagen fibrils in the sub-epithelial region (Fig. 1I,J). Light micrographs of some part of the cornea showed an elevation in the epithelium with a large scar (Fig. 1K). The BW in these region was absent and replaced by collagen fibrils (Fig. 1L).

**Figure 2:**
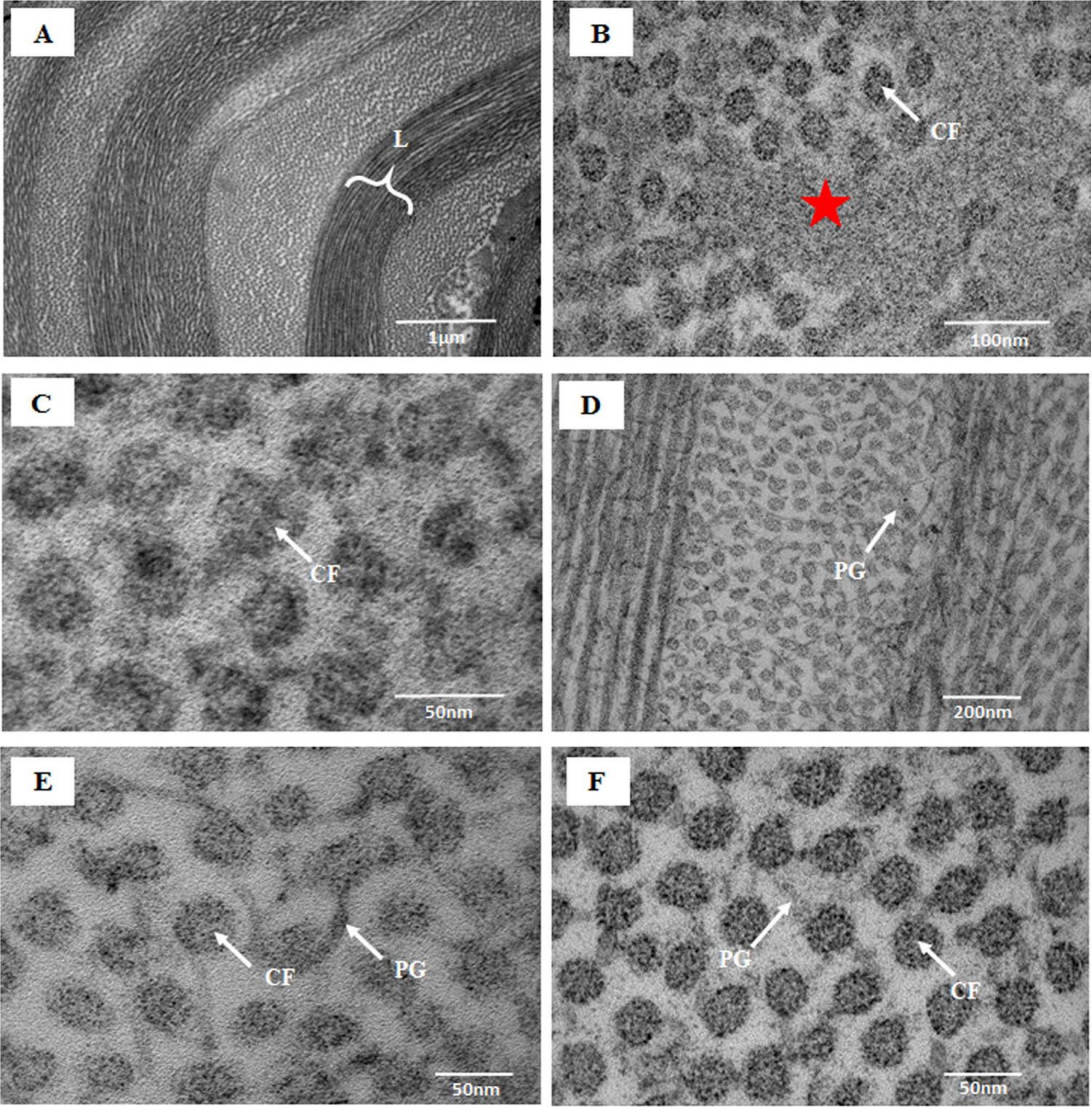
Electron micrographs of the posterior stroma at the periphery of normal and keratoconus corneas; (**A**) Undulating lamellae at the pre-Descemet’s membrane of the KC cornea; (**B**) Electron dens material (red star) in between the degenerated collagen fibrils (CFs) of the undulating lamellae shown in (**A**) (Osmium tetroxide fixation); (**C**) Degenerated CFs at the pre-Descemet’s membrane of the KC cornea (Osmium tetroxide fixation); (**D,E**) Large proteoglycans (PGs) around the CF in the posterior stromal lamellae of KC cornea (Cuprolinic + glutaraldehyde fixation); (**F**) Organised CF connected by PGs in the posterior stroma of the normal cornea. CF = Collagen fibrils, PG = Proteoglycan, L = Undulating lamellae.

The lamellae in the anterior, middle and posterior stroma were degenerated and numerous undulating lamellae were observed at the pre-Descemet’s membrane (DM) area of the periphery of the KC cornea (Fig. 2A). In these undulating lamellae, CFs were degenerated and a large deposits of electron-dense material were observed in between the CFs (Fig. 2B,C). The CFs were electron dense and microfibrils within the CFs were not distinguished from each other (Fig. 2C). Similar findings were observed in the central part of the cornea. These degenerated CFs were attached to each other by abnormally larger proteoglycans (Fig. 2D,E) compared to the PGs of the normal cornea (Fig. 2F).

**Figure 3:**
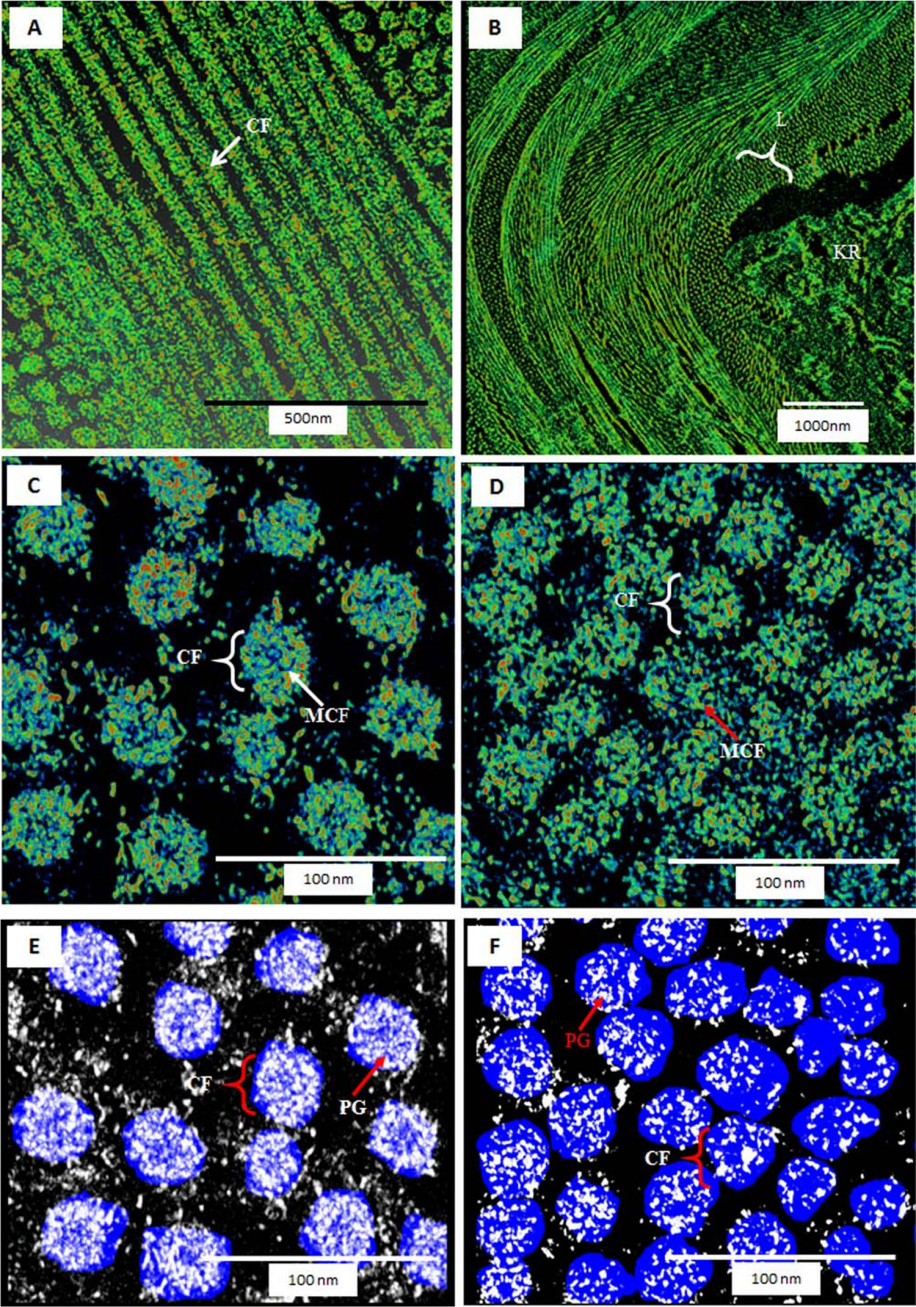
3D transmission electron tomography of posterior stroma at the periphery of normal and keratoconus corneas; (**A**) 3D tomography of the lamellae of the normal cornea (Cuprolinic blue fixation); (**B)** 3D tomography of the undulating lamellae of the KC cornea (Cuprolinic blue fixation); (**C)** 3D tomography of the collagen fibrils (CFs) of the normal cornea showing the micro-fibrillar arrangement within CFs (Cuprolinic blue fixation); (**D**) 3D tomography of the CF of the KC cornea showing the disintegration of the micro-fibrillar arrangement within CFs (Cuprolinic blue fixation); (**E**) 3D tomography of the CFs of the normal cornea showing large number of proteoglycans (PGs) within the CFs (Cuprolinic blue fixation); (**F**) 3D tomography of the CFs of the KC cornea showing small number of PGs within the CFs (Cuprolinic blue fixation). CF = Collagen fibrils, KR = Keratocyte, L = Lamellae, MCF = Microfibrils, PG = Proteoglycan.

3D tomography revealed that the undulating lamellae in the posterior stroma of the KC periphery contained disoriented CFs compared to the normally oriented CF in periphery of the normal cornea (Fig. 3A,B). The keratocytes were crushed in between these undulating lamellae (Fig. 3B). 3D tomography showed that in the periphery of the normal cornea, the microfibrils within the CFs of the posterior stroma were organised (Fig. 3C). In the KC peripheral region, the CFs of the posterior stroma were degenerated and the microfibrils within the CFs were disintegrated and floating around in the posterior stroma (Fig. 3D). Similar findings were observed in the central part of the cornea. 3D tomography also revealed that in the normal posterior stroma of the peripheral region a large number of organised PGs were present around the CFs (Fig. 3E). In the KC posterior stroma of the periphery, the CFs were surrounded by very few PGs (Fig. 3F).

### CF diameter analysis of the normal and KC cornea

**Figure 4:**
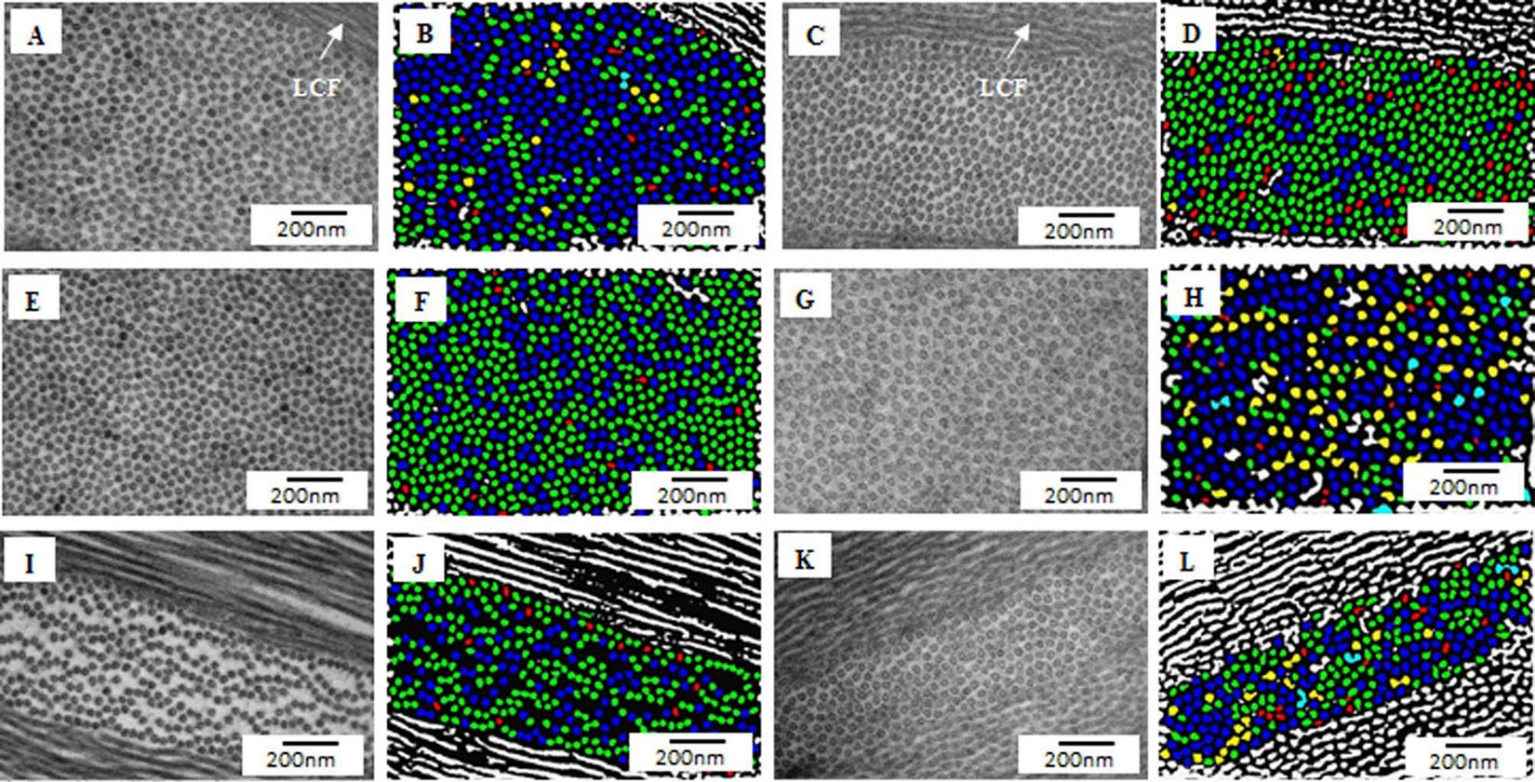
Electron micrographs and digital colour coded images of collagen fibrils (CFs) in the keratoconus cornea; (**A**) Electron micrograph of anterior stroma of central part of the KC cornea; (**B**) Colour coded image of (**A**), containing mostly blue (25–30 nm) and few green (20–25 nm) colour coded CFs (Osmium tetroxide fixation); (**C**) Electron micrograph of anterior stroma of peripheral part of the KC cornea (**D**) Colour coded image of (**C**) containing mostly green (20–25 nm) and few blue (25–30 nm) colour coded CFs (Osmium tetroxide fixation); (**E**) Electron micrograph of middle stroma of central part of the KC cornea; (**F**) Colour coded image of (**E**) containing mixture of green (20–25 nm) and blue (25–30 nm) colour coded CFs (Osmium tetroxide fixation); (**G**) Electron micrograph of middle stroma of peripheral part of the KC cornea; (**H**) Colour coded image of (**G**) containing mostly blue (25–30 nm) and few yellow (30–35 nm) colour coded CFs (Osmium tetroxide fixation); (**I**) Electron micrograph of posterior stroma (PS) of central part of the KC cornea; (**J**) Colour coded image of (**I**) containing mixture of green (20–25 nm) and blue (25–30 nm) colour coded CFs (Osmium tetroxide fixation); (**K**) Electron micrograph of posterior stroma of peripheral part of the KC cornea; (**L**) Colour coded image of (**K**) containing mostly blue (25–30 nm) and few green (20–25 nm) colour coded CFs (Osmium tetroxide fixation). LCF = Longitudinal running collagen fibrils. Red: 15–20 nm; Green: 20–25 nm; Blue: 25–30 nm; Yellow: 30–35 nm; Terracotta: 35–40 nm.

Electron micrographs of the anterior, middle and posterior stroma of the normal and KC cornea (center and periphery) were processed into color coded digital images to analyse the collagen fibril diameter and interfibrillar spacing (Fig. 4, Table 1). The mean fibrillar diameter of the anterior, middle and posterior of both the center and the periphery of the KC cornea, were significantly smaller (p < 0.001) compared to the mean fibrillar diameter of the anterior, middle and posterior of the normal cornea (Table 1).

**Table 1:** Comparison of collagen fibril diameter and interfibrillar spacing of anterior, middle and posterior stroma between Keratoconus and normal corneas, and within the same KC or normal cornea.

Stromal Zone	Corneal Area	Collagen Fibril Diameter (Mean ± SE)		Interfibrillar spacing (Mean ± SE)	
		KC (n = sample size)	Normal (n = sample size)	KC (n = sample size)	Normal (n = sample size)
Anterior stroma	Center	27.08 ± 0.08^*^ (n = 4)	32.13 ± 0.09 (n = 4)	37.89 ± 0.11^*^ (n = 4)	44.26 ± 0.16 (n = 4)
	Periphery	26.76 ± 0.07^*, †^ (n = 4)	31.43 ± 0.73^†^ (n = 4)	39.23 ± 0.11^*,†^ (n = 4)	46.89 ± 0.11^†^ (n = 4)
Middle stroma	Center	27.33 ± 0.06^*^ (n = 4)	30.68 ± 0.08 (n = 4)	38.02 ± 0.08^*^ (n = 4)	50.93 ± 0.10 (n = 4)
	Periphery	27.47 ± 0.06^*,†^ (n = 4)	31.48 ± 0.06^†^ (n = 4)	38.32 ± 0.08^*,†^ (n = 4)	49.98 ± 0.12^†^ (n = 4)
Posterior stroma	Center	25.94 ± 0.06^*^ (n = 4)	30.29 ± 0.08 (n = 4)	35.22 ± 0.11^*^ (n = 4)	52.23 ± 0.12 (n = 4)
	Periphery	27.19 ± 0.07^*,†^ (n = 4)	30.69 ± 0.10^†^ (n = 4)	37.19 ± 0.11^*,†^ (n = 4)	54.98 ± 0.22^†^ (n = 4)
Mean	Center	26.82 ± 0.04^*^ (n = 4)	30.87 ± 0.05 (n = 4)	37.08 ± 0.06^*^ (n = 4)	49.92 ± 0.08 (n = 4)
	Periphery	27.17 ± 0.04^*,†^ (n = 4)	31.24 ± 0.04^†^ (n = 4)	38.22 ± 0.06^*,†^ (n = 4)	50.36 ± 0.09^†^ (n = 4)

*Significant difference (p < 0.001) compared with corresponding zone in the normal cornea (Mann-Whitney test).

^†^Significant difference (p < 0.001) compared with central area in the same cornea (KC or normal) (Mann-Whitney test).

SE: Standard error.

Within the KC cornea, the mean CF diameter of the central-anterior stroma was significantly (p < 0.001) larger than the peripheral-anterior stoma whereas the central-middle and posterior CF diameter was significantly (p ˂ 0.001) smaller than the peripheral-middle and posterior stroma (Table 1).

**Figure 5:**
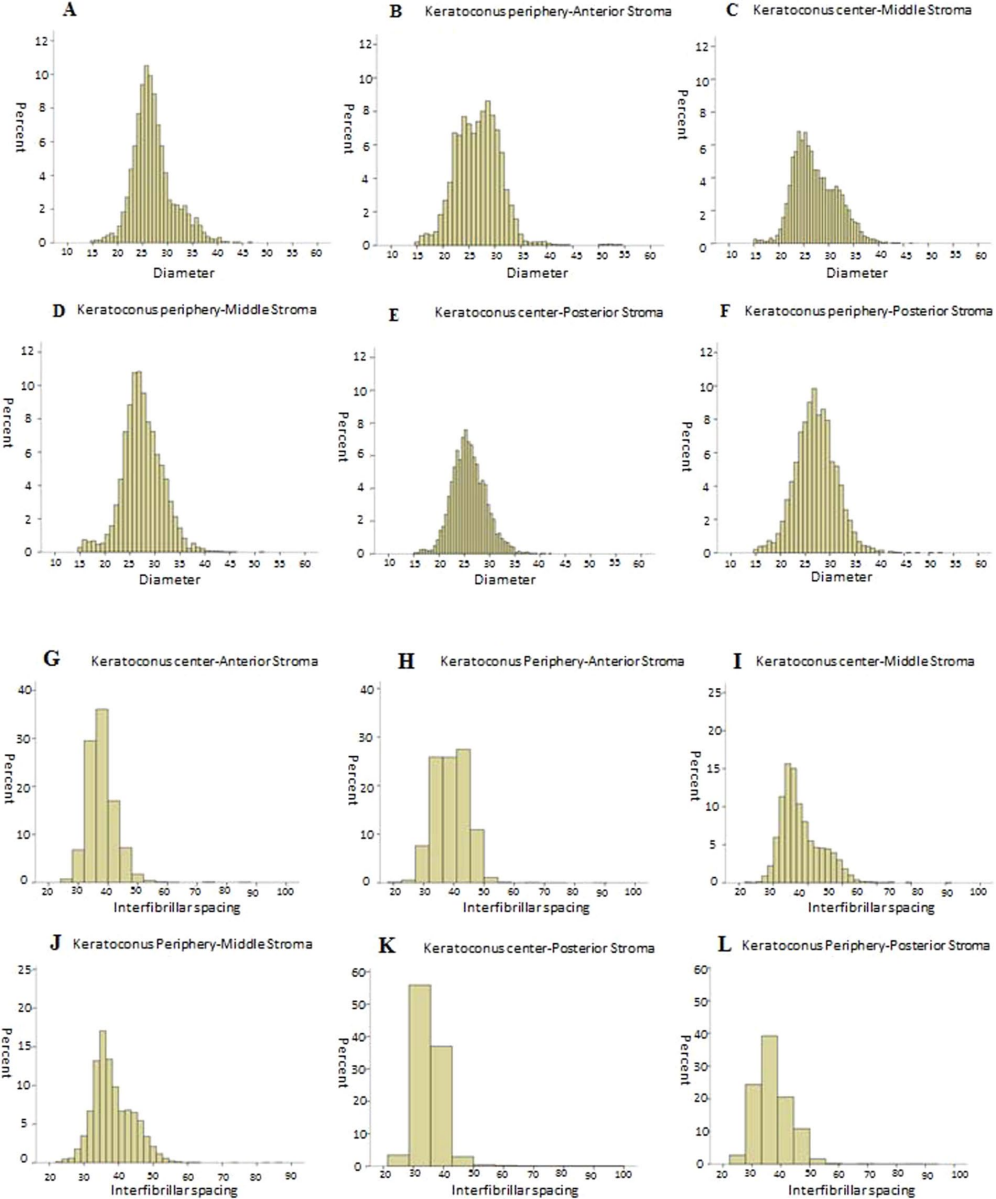
Frequency distribution of collagen fibrils diameters (nm) in Keratoconus cornea; (**A**) Anterior stroma at the center; (**B)** Anterior stroma at the periphery; (**C**) Middle stroma at the center; (**D**) Middle stroma at the periphery; (**E**) Posterior stroma at the center; (**F**) Posterior stroma at the and periphery. Frequency distribution interfibrillar spacing (nm) in the keratoconus cornea; (**G**) Anterior stroma at the center; (**H**) Anterior stroma at the periphery; (**I**) Middle stroma at the center; (**J**) Middle stroma at the periphery; (**K**) Posterior stroma at the center, (**L**) Posterior stroma at the periphery.

In the normal cornea, the majority of the fibrils in the center as well as in the periphery are ≥30 nm in diameter. In the centre of KC cornea, the majority of the fibrils at the anterior (51.02%), middle (38.57%) and posterior stroma (48.95%) have a diameter ranging from 25–30 nm. This was also the case for the fibrils in the periphery of KC anterior (41.78%), middle (50.77%) and posterior stroma (47.85%). However, the variability in fibrillar diameter seems to be more in the periphery rather than the center with higher percentage of fibrils <20 nm (Fig. 5). There was no significant inter-sample and inter-group variability of the CF diameter of both KC and normal cornea.

### Interfibrillar spacing in KC and normal cornea

The mean interfibrillar distance in the anterior, middle and posterior stroma of both the center and periphery of the KC cornea was significantly smaller (p < 0.001) than the mean interfibrillar distance in the anterior, middle and posterior stroma of the normal cornea Table 1. On comparing the inter-fibrillar distance within the same cornea, there was significant difference (p < 0.001) between the centre and periphery of both the KC and normal cornea throughout the stroma (Table 1).

In the normal cornea, the majority of the fibrils in the center as well as the periphery have an interfibrillar distance that ranges between 40–50 nm. In the center of KC cornea, the majority of the fibrils at the anterior (70.7%), middle (66.4%) and posterior stroma (81.6%) have a center-center interfibrillar distance ranging between 30–40-nm. Likewise, in the periphery of KC cornea the majority of the fibrils in the anterior (49.9%), middle (62.3%) and posterior stroma (62%) were also 30–40 nm apart (Fig. 5). There was no significant difference of interfibrillar spacing within sample and within group both KC and normal cornea.

### Proteoglycans Area and density

**Figure 6:**
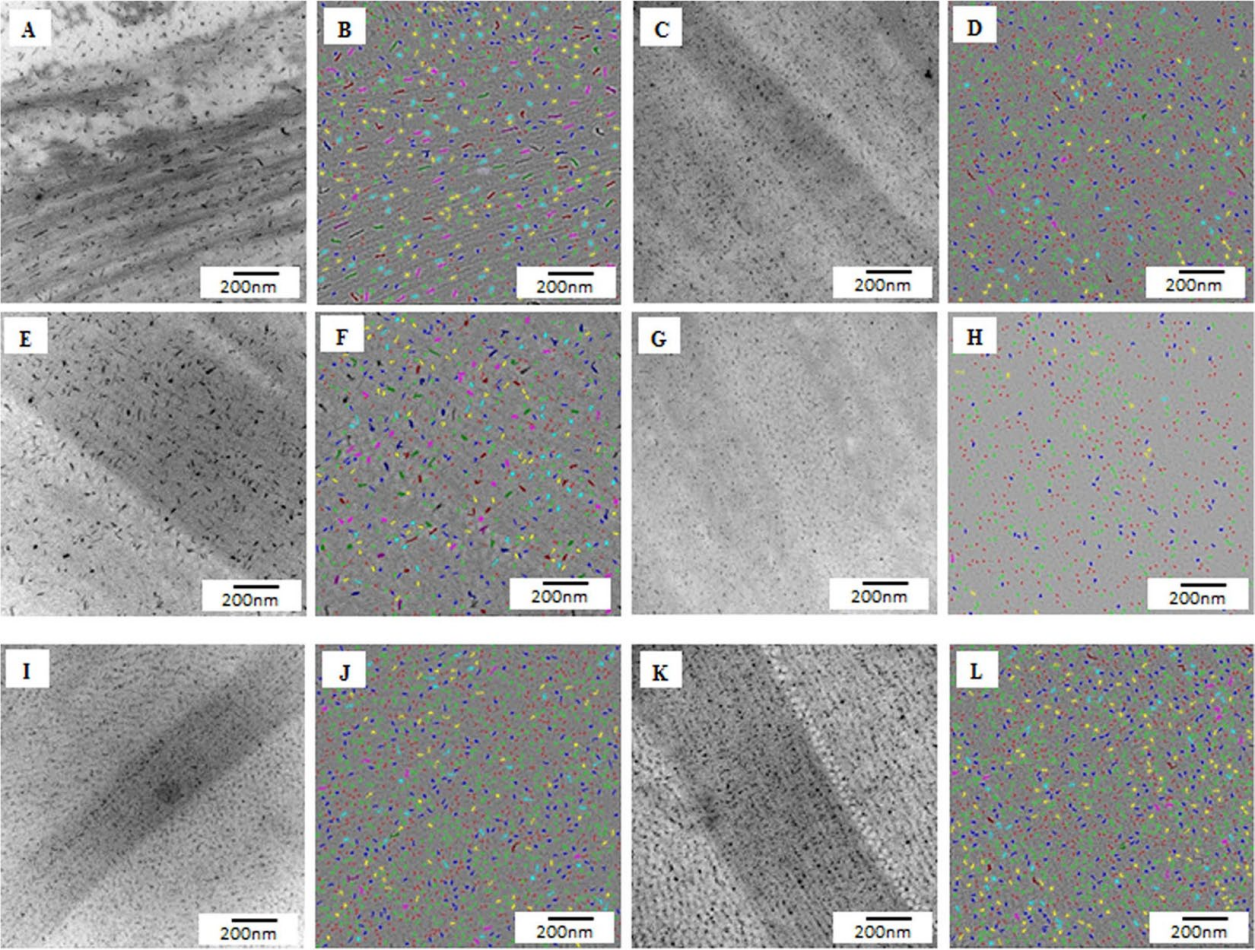
Electron micrographs and digital colour coded images of proteoglycans in the keratoconus (KC) cornea; (**A**) Electron micrograph of anterior stroma (AS) of central part of the KC cornea; (**B**) Colour coded image of (**A**); (**C**) Electron micrograph of AS of peripheral part of the KC cornea; (**D**) Colour coded image of (**C**); (**E**) Electron micrograph of middle stroma (MS) of central part of the KC cornea; (**F**) Colour coded image of (**E**); (**G**) Electron micrograph of MS of peripheral part of the KC cornea; (**H**) Colour coded image of (**G**); (**I**) Electron micrograph of posterior stroma (PS) of central part of the KC cornea; (**J**) Colour coded image of (**I**); (**K**) Electron micrograph of PS of peripheral part of the KC cornea; (**L**) Colour coded image of (**K**). Red: 30–75 nm^2^; Green: 75–120 nm^2^; Blue: 120–165 nm^2^; Yellow: 165–210 nm^2^; Terracotta: 210–255 nm^2^, Pink: 255–300 nm^2^; Brown: 300–345 nm^2^; Olive: 345–390 nm^2^; Dark blue: 390–435 nm^2^, Purple: 435–480 nm^2^.

To analyse the PGs area and density of normal and keratoconus cornea, the digital images of the anterior, middle and posterior stroma (center and periphery) were processed into color coded digital images (Fig. 6). The mean PGs area of the anterior, middle and posterior stroma of the central KC were significantly (P < 0.0001) larger than the mean PGs area of the anterior, middle and posterior stroma of the central part of the normal cornea Table 2. In the peripheral cornea, the anterior stromal PGs area of the KC was larger than the normal PGs area, whereas in the middle stroma, the PGs area of KC was smaller (P < 0.0001) than the normal PGs area. There was no significant difference in the posterior stroma (Table 2). The mean PGs area of the anterior, middle and posterior stroma in the center was significantly (P < 0.0001) larger than the mean PGs area of the anterior, middle and posterior stroma of the periphery within the KC cornea (Table 2) (Fig. 6). Within the normal cornea, there was no difference in the PGs area of the anterior stroma between the central and peripheral areas.

**Table 2:** Comparison of Proteoglycans area and density of anterior, middle and posterior stroma between Keratoconus and normal corneas, and within the same KC or normal cornea.

Stromal Zone	Corneal Area	Distribution of Proteoglycans with Different Areas				Proteoglycan Area in nm^2^ (Mean ± SE)	Proteoglycan Density/μm^2^	CV
		< 75 nm^2^	75–119.99 nm^2^	120–164.99 nm^2^	≥165 nm^2^			
Anterior stroma	KC Center	31.9%	34.2%	18.9%	15.0%	111.89 ± 0.80^*^ (n = 4)	452	24
	Normal Center	45.3%	32.1%	14.2%	8.4%	91.61 ± 0.69 (n = 4)	203	12
	KC Periphery	38.2%	37.7%	14.9%	9.2%	99.22 ± 0.60^*,†^ (n = 4)	387	38
	Normal Periphery	44.4%	37.3%	13.0%	5.4%	90.54 ± 0.46 (n = 4)	447	17
Middle stroma	KC Center	26.9%	32.7%	17.9%	22.4%	126.43 ± 0.84^*^ (n = 4)	455	25
	Normal Center	49.6%	30.8%	12.6%	7.0%	86.80 ± 0.66 (n = 4)	181	9
	KC Periphery	48.3%	38.0%	10.3%	3.4%	85.97 ± 0.47^*,†^ (n = 4)	389	33
	Normal Periphery	39.8%	37.4%	15.5%	7.3%	96.01 ± 0.48^†^ (n = 4)	446	13
Posterior stroma	KC Center	34.8%	37.6%	16.3%	11.3%	104.87 ± 0.60^*^ (n = 4)	520	28
	Normal Center	51.6%	27.8%	12.2%	8.4%	87.39 ± 0.68 (n = 4)	192	8
	KC Periphery	39.7%	38.4%	14.0%	7.9%	96.40 ± 0.48^†^ (n = 4)	493	56
	Normal Periphery	39.1%	37.4%	16.0%	7.5%	96.76 ± 0.46^†^ (n = 4)	482	19

*Significant difference (p < 0.0001) compared with corresponding zone in the normal cornea (Mann-Whitney test).

^†^Significant difference (p < 0.0001) compared with central area in the same cornea (KC or normal) (Mann-Whitney test).

SE: Standard error.

CV: Coefficient of variation.(variability in density).

#### PGs density analysis

An assessment of PGs density in the center of the KC cornea revealed that the PGs density in the anterior (452/μ^2^), middle (455/μ^2^) and posterior stroma (520/μ^2^) was higher than the PGs density in the anterior (203/μ^2^), middle (181/μ^2^) and posterior stroma (192/μ^2^) in the center of the normal cornea Table 2. In contrast, the PGs density in the anterior (387/μ^2^) and middle stroma (389/μ^2^) in the periphery of the KC cornea was lower than that in the anterior (447/μ^2^) and middle stroma (446/μ^2^) and in the periphery of the normal cornea (Table 2). Moreover, the peripheral stroma in the KC cornea showed the highest variability in PGs density (CV ranging between 33–56) with areas of extremely high density and others of extremely low density (Table 2). There was no significant difference of PGs area and density within sample and within groups of both KC and normal cornea.

## Discussion

The lamellae in the posterior stroma at the periphery of the KC cornea were degenerated and numerous undulating lamellae were emerging out from the DM as previously reported^17^. The present study revealed that the CFs of these undulating lamellae were degenerated and disorganised. 3D electron tomography showed that the microfibrils within the CFs of these lamellae were disintegrated and randomly spread in the interfibrillar space. 3D tomography also showed the degeneration of the proteoglycans around, and in between, the undulating lamellae of the posterior stroma at the periphery of the KC cornea.

Quantitative analysis of the CFs showed a significant reduction in fibril diameter and in ‘center-to-center spacing’. We believe that the thinning and close packing of CFs in the central cornea correlates well with corneal thinning in the KC cornea which has been reported in the literature^7,15,21^. The PGs density and area in the central as well as the peripheral cornea differed significantly from those observed in the normal cornea. Our observations of alterations in the CF and PGs in the central cornea are consistent with previous reports^9–14^. The changes in the CFs and PGs in the peripheral cornea have not been reported previously. Our novel observation of the alteration of the CFs and PGs in the periphery of the KC cornea suggests the involvement of peripheral stroma in the disease process.

Fibril growth is regulated by the core proteins of the proteoglycans decorin, lumican and keratocan and changes in their synthesis are associated with changes in fibril diameter^22^. The interfibrillar distance on the other hand, is regulated by the GAG side chains through stromal hydration regulation and the interaction with adjacent fibrils^22^. So while the protein cores attach to the CFs, the GAGs extend to fill the space between adjacent fibrils. *In vitro* analysis of the fibril diameter revealed that the absence of GAG side chains of lumican and decorin results in the formation of very thin fibrils^23^. Funderburgh *et al*.^10^ suggested that that Keratan Sulphate (KS)-PGs in the KC cornea contained fewer, or shorter, sulphated KS chains with unaltered expression of the KS core protein. We believe that alterations in the sulphated KS side chains result in over activity of the core protein and thus formation of thinner CFs in the KC cornea. Moreover, with the interfibrillar space filled with shorter GAG chains, the fibrils come closer together and thus the distance is reduced.

Our observation of reduced PGs density in the anterior and middle stroma of the KC cornea in comparison to the normal cornea correlates well with the reduced PG expression and KS staining previously reported in the center of the KC cornea^10,12–14^. Akhtar *et al*.^12^ reported that in severe KC the density of KS-PGs in the central cornea reduce significantly in comparison to early onset of KC and the normal cornea. Our observation of the profound diminution of PGs density in the peripheral stroma suggests that it was subject to severe disease alterations, which indicates that it has an early involvement in the disease process. The PGs area in the peripheral posterior stroma of our KC corneas (96.40 nm^2^) was similar to the PGs area of the peripheral posterior stroma of the normal cornea (96.76 nm^2^). However, in this particular area of the KC cornea, a large variability of PGs density with focal areas of very low and of very high density, was observed. Accordingly, we believe that this area was subject to extreme alterations in the PG synthesis and expression.

Akhtar *et al*.^9^ reported a significant increase in PGs density and mean area within the center of the KC cornea compared with the normal cornea. Consistent with their findings, our findings in the KC cornea showed that the PGs at the central part of the KC cornea were consistently larger in area than those in the central part of the normal cornea in all stromal zones. Sawaguchi *et al*.^11^ reported a significant increase in large filaments of dermatan Sulfate (DS)-PGs particularly in scarred areas in the KC cornea. Since those filaments resemble those observed in other scarred tissue, Sawaguchi *et al*.^11^ suggested that their presence in the KC cornea is secondary to scar formation. In light of his suggestion, we believe that the higher PG density in the central area of the KC cornea reflects alterations which are secondary to cone progression rather than relating to primary disease changes. Our observation of an increased density of larger PG filaments in the central area of the KC cornea supports this conclusion.

The alterations in PG expression in the KC cornea help to explain the disruption of the normal lamellar outline observed in the KC cornea as well as alterations in CFs diameter and the spacing in the peripheral cornea. These alterations were also observed in the central corneas in our study and in previous reports^9,24,25^.

## Conclusion

Our 3D imaging and quantitative analysis of CFs and PGs revealed the disruption in the distribution of PGs in the undulating lamellae just above the DM in the peripheral cornea. These findings suggest that in the KC cornea, the degeneration of the PGs around the CFs occurred leading to breakage of the CFs and degeneration of the microfibrils within the CFs. This results in a reduction of the CFs diameter. Consequently, the lamellae which are composed of these degenerated CFs were biomechanically weak and prone to disorganization and undulation which eventually alter the curvature of the cornea or cone formation.
